# Fluorescent Dyes in Hydrological Tracing: Application Methods, Ecotoxicological Effects, and Safe Application Levels

**DOI:** 10.3390/jox16020045

**Published:** 2026-03-03

**Authors:** Carlos J. A. Campos, Louis A. Tremblay, Olivier Champeau, Gregory Goblick

**Affiliations:** 1 Jacobs, 47 Hereford Street Level 2, Wynn Williams Building, Christchurch 8013, New Zealand; carlos.campos@wrcgroup.com; 2 Water Research Centre Ltd., Frankland Road, Blagrove, Swindon SN5 8YF, Wiltshire, UK; 3 New Zealand Institute for Bioeconomy Science Ltd., Port Nelson, Nelson 7010, New Zealand; 4 Environmental Protection Authority, Wellington 6011, New Zealand; olivier.champeau@epa.govt.nz; 5 Human Foods Program, Office of Laboratory Operations and Applied Science, US Food and Drug Administration, Dauphin Island, AL 36528, USA; gregory.goblick@fda.hhs.gov

**Keywords:** hydrology, ecotoxicology, contaminant tracing, risk assessment, safe application thresholds, rhodamine

## Abstract

Fluorescent dyes are commonly used as tracers in hydrological investigations to quantify transport pathways, residence times, mixing behavior, and connectivity in surface water, groundwater, and coastal systems. Despite their long history of application, the ecological implications of deliberate dye releases are not well understood. This review synthesizes current knowledge on the physico-chemical characteristics, environmental behavior, and ecotoxicological effects of major dye classes, with emphasis on rhodamines, fluorescein derivatives, and sulfonated xanthene dyes commonly used in water tracing studies. Toxicity data for algae, cyanobacteria, invertebrates, and fish show large inter-specific variability. Some dyes, particularly rhodamine B and eosin Y, show acute or sub-lethal effects at concentrations detected during poorly controlled applications. By contrast, dyes with high polarity and extensive sulfonation (e.g., rhodamine WT, sulforhodamine B, pyranine, and fluorescein) show consistently low toxicity and minimal bioaccumulation potential. Environmental fate processes, including photolysis, sorption, and transformation into potentially more reactive products, influence exposure dynamics, especially in clear, shallow, or slow-moving systems. This review also evaluates regulatory frameworks and operational guidance for safe use, identifies gaps in toxicological and fate data, and proposes recommendations for minimizing environmental impact through dye selection, mass optimization, injection design, and monitoring. The findings support the continued use of fluorescent dyes but highlight the need for more systematic assessment of transformation products, chronic and sub-lethal responses, and cumulative exposure in sensitive environments.

## 1. Introduction

Fluorescent dyes have been used for more than a century as diagnostic tools in environmental hydrology, serving as synthetic analogs for tracking water movement and solute transport in natural and engineered systems [1,2,3,4,5]. The first documented dye tracing experiment was conducted in the Danube River catchment in 1877 [6], initiating the development of techniques that now underpin a range of hydrological investigations. When released into surface waters, groundwater systems, or fractured rock, fluorescent dyes enable identification of flow paths, quantification of travel times, and estimation of dispersion and dilution processes with high analytical sensitivity [3,4,7,8]. These data are essential for predicting contaminant plume migration from sources such as wastewater treatment plant discharges, septic systems, landfills, industrial discharges, and agricultural runoff and for informing pollution mitigation and risk assessment strategies [3,4].

Despite their long history and utility, the intentional release of fluorescent dyes has raised ecological and regulatory concerns [8,9,10]. Although dyes are typically applied at very low concentrations (µg/L to mg/L), their suitability as tracers depends on characteristics such as resistance to photodegradation, sorption, and biodegradation; these can also promote environmental persistence [10,11,12]. Early tracer studies often assumed dyes to be inert. However, subsequent ecotoxicological research has demonstrated potential adverse effects in aquatic organisms at concentrations overlapping with or moderately exceeding those detected during field applications [9,13]. Consequently, it is increasingly acknowledged that dye tracers are not universally harmless.

Several factors contribute to uncertainty regarding the ecological safety of fluorescent dyes. Reported toxicity thresholds vary widely among studies due to differences in exposure duration, test organism life stage, water chemistry, and dye purity. Regulatory frameworks often rely on acute lethality endpoints which do not capture sub-lethal responses such as impaired reproduction, altered behavior, oxidative stress, or genotoxicity. These effects have been observed in laboratory studies at concentrations relevant to dye tracing studies [9,14]. The influence of dyes on microbial communities, biofilm processes, and hyporheic zone function is also largely unexplored, despite their central role in nutrient cycling and ecosystem stability. Degradation products, particularly those formed through photolysis or redox transformations, are rarely monitored in field studies, even though their toxicological profiles may differ from those of parent compounds.

These scientific uncertainties are aggravated by substantial variation in national and regional guidance that regulates dye applications. Jurisdictions differ in allowable dye concentrations, acceptable release frequencies, recommended tracer compounds, and monitoring requirements [15]. This results in inconsistent levels of environmental protection. Given these issues, it is essential to improve integration of hydrological benefits of fluorescent dyes with ecological safeguards.

This review synthesizes the ecotoxicological evidence for major tracers used in hydrology, including rhodamines, fluoresceins, pyranine, and related dyes. Specifically, it aims to

- Assess the toxicological effects of fluorescent dyes on exposed aquatic biota in the area where the tracers are applied.
- Establish evidence-based threshold concentrations above which effects are likely to occur and principles for environmentally protective use.
- Identify critical knowledge gaps requiring targeted research.

The overall goal is to inform guidance that supports the responsible use of fluorescent dyes, maintaining their essential role in hydrological investigation and ensuring that applications remain compatible with ecosystem health.

## 2. Overview of Fluorescent Dyes in Hydrological Tracing

### 2.1. Major Classes and Key Characteristics

Fluorescent dyes used in hydrological tracing fall predominantly into three structural classes: xanthenes, antraquinones, and aminonaphthalenesulfonates [1,11]. Their chromophore structures determine fluorescence intensity, hydrophilicity, photostability, and chemical reactivity, which in turn influence their suitability as hydrological tracers and their potential ecological effects. Table 1 summarizes the main physico-chemical characteristics of representative dyes used in water tracing studies.

Xanthene dyes, including rhodamines, fluoresceins, and eosins, are the most widely used hydrological tracers because of their strong fluorescence quantum yields, structural stability, strong solubility, and susceptibility to fluorometric detection [10]. Among these, rhodamine WT is the preferred tracer for surface water studies because of its high photostability, comparatively low sorption to mineral and organic substrata, and emission in the orange-red region, which reduces interference from natural background fluorescence [7]. Sutton et al. demonstrated that rhodamine WT comprises two structural isomers with distinct sorption characteristics and slightly different emission spectra; these structural differences can introduce variability in measurements, particularly in porous media [16].

Rhodamine B and rhodamine 6G share similar optical properties [17] but adsorb more strongly onto organic matter and degrade more readily under sunlight than rhodamine WT [7,8]. Sulphonated derivatives such as sulforhodamine B and eosin show strong sorption onto mineral surfaces, making them unsuitable as conservative tracers in saline or mineral-rich groundwater, where sorption processes are enhanced [7,18]. Eosin Y, a brominated fluorescein derivative, has strong absorption in the visible range but photodegrades rapidly, generating reactive intermediates and photoproducts [7,19].

Fluorescein sodium (uranin) has strong green fluorescence, high solubility, and relatively low sorption onto mineral surfaces [7,19]. However, fluorescein is very sensitive to photobleaching and oxidative degradation, particularly under acidic conditions (pH < 6) or in oxygenated, sunlit waters [8]. These characteristics make fluorescein suitable for short-term studies in shaded or subsurface environments but less appropriate for long-range surface water applications. In moderately saline groundwater, fluorescein sorption increases, which can change its conservative behavior relative to rhodamines [18].

Pyranine fluoresces in the blue-green range and is strongly pH-dependent because of its phenolic group, making it useful for the simultaneous monitoring of solute transport and pH [8].

Sodium naphthionate is less commonly used but can be advantageous in colored or organic-rich waters where xanthene fluorescence is masked [18]. It emits in the violet-blue region and is more resistant to microbial degradation than fluorescein derivatives. However, under reducing conditions, it may transform into aromatic amines of potential toxicological concern [20].

Overall, the combination of detectability, solubility, photostability, and low reactivity determines the hydrological utility of these dyes. These properties also influence ecotoxicity.

### 2.2. Applications

Fluorescent dyes are used in a broad range of hydrological investigations, including surface water hydrodynamics, groundwater flow and transport, and water exchange between catchment compartments [3,4,11,21,22]. Their ability to provide high-resolution spatial and temporal information allows for the quantification of hydraulic connectivity, contaminant dispersion, residence times, and solute-transport processes.

In rivers, estuaries, channels, and coastal environments, dye tracers are used to quantify flow velocities, dispersion coefficients, and mixing patterns [5,23,24,25,26]. Released dyes generate breakthrough curves that support advection–dispersion modeling and calibration of transport models. Dyes also support operational applications, including mapping piscicide plumes during fishery management [27].

In groundwater systems, particularly karst aquifers, dyes are used to delineate flow paths, identify hydraulic boundaries, and quantify travel times [28,29,30,31,32]. In a tetrachloroethylene/dye column test study, the authors concluded that multi-tracer approaches using rhodamine WT, sulphorhodamine B, and eosin were possible candidates as partitioner tracers to allow for the simultaneous mapping of multiple flow connections [33]. But these were laboratory-controlled experiments and have not been tested in the field.

In urban settings, dyes are used to assess sewer connectivity [34,35], identify inflow and infiltration [36], and characterize mixing in wastewater treatment wetlands [37,38] and treatment plants [39,40]. These applications often involve repeated or continuous injections, requiring careful dose control to limit ecological exposure.

### 2.3. Release Methods and Data Analysis

**Figure 1:**
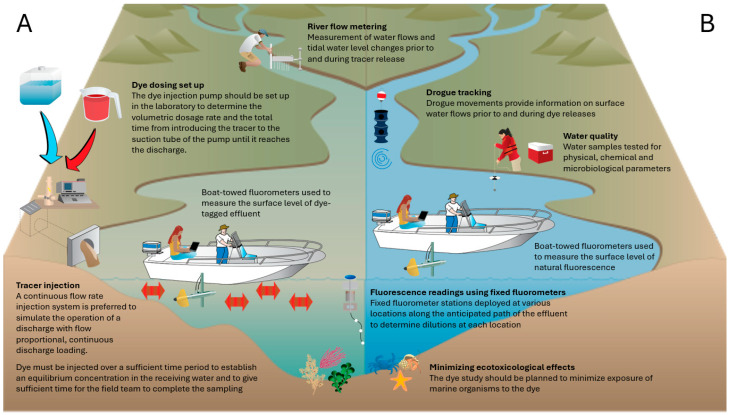
Conceptual model of a field study involving the continuous release of a fluorescent tracer. (**A**) field activities during tracer release; (**B**) field activities prior to tracer release.

Dye release methods are selected according to hydrological objectives, site characteristics, and regulatory constraints [41]. Figure 1 summarizes typical activities associated with continuous tracer releases, including preparatory assessments and field operations. Two release configurations are commonly used:

- Pulse (slug) injection: It involves the introduction of a known mass of dye at once, producing a transient concentration wave that propagates downstream or through subsurface media. Breakthrough curves from pulse injections show a rapid rise to peak concentration followed by a long, dispersive tail controlled by advection, dispersion, and sorption. Pulse injections are widely used to determine travel times, dispersion coefficients, and hydraulic connectivity [2,3,12].
- Continuous injection: It involves metering dye at a controlled rate to achieve quasi-steady-state conditions at a monitoring point. They support discharge estimation, dilution gauging, and mixing assessments [2,3,42]. Continuous injections require pump systems, redundancy, and safeguards against overdosing, particularly in low-flow systems, where concentration excursions can occur rapidly.

The advantages and limitations of each method are summarized in Table 2. From an ecotoxicological perspective, pulse injections create short-duration, high-concentration exposure near the release point, while continuous injections maintain lower concentrations over longer periods. Environmental conditions such as shading, turbidity, and dissolved organic carbon influence dye persistence by modifying sorption and photolysis rates [11].

**Figure 2:**
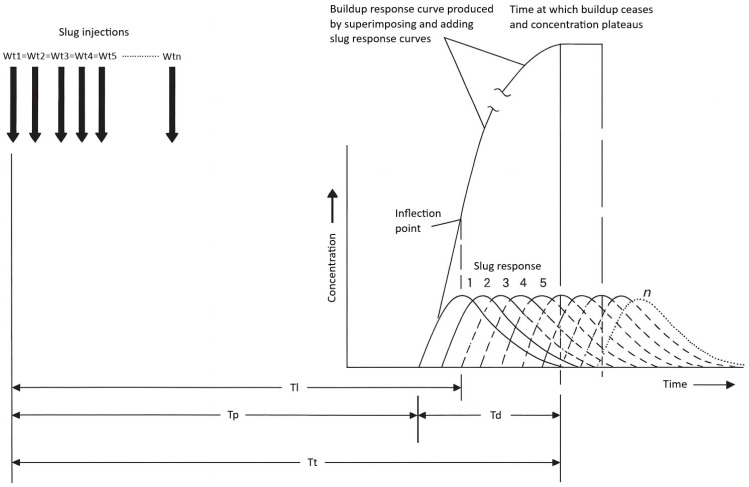
Conceptual model of dye time–concentration curves for slug and constant injections recorded at three points across a channel at three distances downstream from the injection point. Reproduced from Kilpatrick (1992) [2]; credit: U.S. Geological Survey, Department of the Interior/USGS. Wt = weight of tracer injected; Tl = elapsed time to leading edge of the dye plume; Tp = elapsed time to peak dye concentration; Td = time for dye cloud to pass any one point in a section; Tt = elapsed time to trailing edge of the dye plume.

Dye response curves vary across a channel due to differences in lateral velocity; centerline profiles show earlier and higher peaks, while near-bank profiles are delayed and broadened. Kilpatrick and Cobb [3] proposed a linear superposition method that conceptualizes a continuous injection as the sum of an infinite series of small, time-shifted slug injections (Figure 2). By summing these increments, the method predicts the time required for downstream concentrations to stabilize. Convergence of successive cumulative curves indicates the attainment of steady state. This approach enables robust design of continuous injection studies without extensive preliminary field trials [3].

Dye concentration data from fixed stations, boat-mounted sensors, or autonomous platforms can be interpolated to produce synoptic maps of dye distribution at successive time steps [43,44]. Mobile fluorometry, using sensors mounted on boats, kayaks, jetskis, or autonomous vehicles, allow for high-frequency transect sampling and detailed characterization of plume structure, mixing conditions, and lateral variability [45,46,47,48]. When georeferenced with GPS, these measurements support the validation of hydrodynamic models and the identification of coastal recirculation zones or channel-scale heterogeneity [49,50].

**Figure 3:**
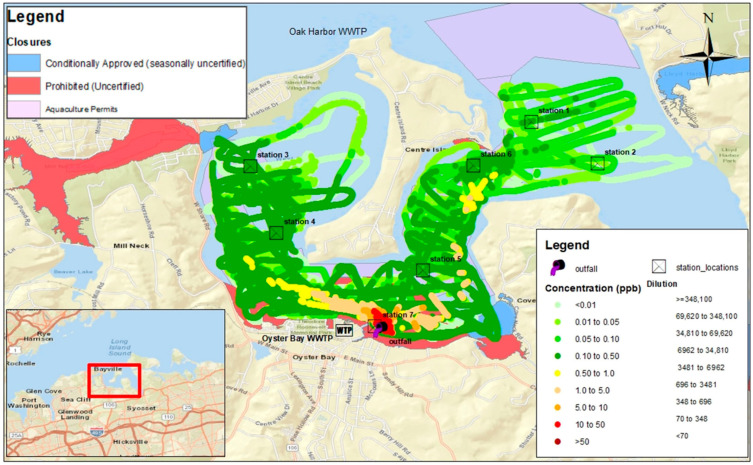
Map of rhodamine WT dye concentrations recorded by boat-towed fluorometer in Oyster Bay estuary, New York (USA), downstream of the Oyster Bay Wastewater Treatment Plant outfall in a study conducted between 29 November and 1 December 2023. Colored estuarine areas show classification of shellfish growing areas. Cross mark boxes show location of fixed fluorometers. Map generated by RAFT-MAP software. Source: [51].

Advanced data collection and visualization systems have been developed to support the real-time monitoring and analysis of dye tracer studies. The FDA’s RAFT-MAP (Real-time Aquatic Fluorescence Tracking and Mapping Application Platform) is a mobile Geographic Information System (GIS) specifically designed for collecting, visualizing, and analyzing field data in real time during fluorescent dye studies. This software platform integrates GPS positioning with fluorometric measurements to generate georeferenced concentration maps and support immediate assessment of plume evolution and mixing dynamics. Such systems enhance the ability to monitor environmental exposure in real time and adjust study protocols to maintain concentrations within safe limits [43]. Mapped concentration fields such as those shown in Figure 3 can reveal residence time gradients, stratification effects, and bathymetric influences on plume evolution. When combined with continuous time-series data, spatial mapping provides a robust basis for estimating transport rates, assessing near-field exposure dynamics, and identifying environmentally sensitive areas potentially affected during tracer studies [43].

## 3. Toxicological Effects on Aquatic Organisms

Fluorescent dyes are widely regarded as operationally safe when used at concentrations typical of hydrological studies. Fluorescent dyes have low log Kow, as a requirement for an effective dye is that it remains in the water column and exhibits low adsorption onto organic substrate material [52]. For instance, the log Kow values of the commonly used fluorescent dye tracers fluorescein and eosin Y were reported at <2.8 and negatively correlated to pH [52]. Environmental fate studies on dyes confirm that hydrophilicity and electrostatic interactions with water and surfaces inhibit sorption and partitioning into organisms, reducing bioaccumulation (e.g., adsorption behavior depends strongly on dye charge and hydrophilicity) [53]. Studies on ionic liquids show that lipophilicity is one of the main drivers of accumulation and toxicity, while highly hydrophilic or strongly ionic compounds exhibit low affinity for biological membranes and therefore lower bioaccumulation potential [54]. However, this perception is largely based on absence of observed ecological effects rather than by comprehensive toxicological evidence. A working group of the German Federal Environmental Agency addressed the uncertainty of the negative effects of chemical tracers and concluded that based on toxicity and ecotoxicity data and their expert judgement, many chemicals are safe but that further assessment should be considered prior to their application to water bodies [13]. Laboratory studies show substantial variability in species sensitivity, reflecting differences in exposure conditions, dye purity, and environmental parameters such as light, pH, and organic matter content. This section summarizes the available ecotoxicological data across major groups of aquatic organisms, highlights mechanisms of toxicity, compares species sensitivity, and reports implications for environmentally protective tracer applications. Table S1 summarizes data on toxicity of fluorescent dyes to aquatic organisms reported in the literature [12,14,20,55,56]. Key information is provided in the next subsections.

### 3.1. Microalgae and Cyanobacteria

Microalgae are consistently among the most sensitive taxa to fluorescent dyes [9,57,58]. Recent standardized tests (e.g., green algae *Raphidocelis subcapitata* and *Chlorella vulgaris*) show that rhodamine B can inhibit algal growth at concentrations below those affecting many invertebrates and fish (Table S1). In particular, the sensitivity of *R. subcapitata* highlights the susceptibility of photosynthetic organisms to dyes that alter light absorption or generate reactive intermediates during photolysis [58]. Sodium naphthionate and other strongly sulfonated dyes exhibit substantially lower algal toxicity, consistent with their limited membrane permeability and minimal cellular uptake.

Mechanistically, toxicity in algae is associated with light attenuation, which reduces photosynthetically active radiation, oxidative stress linked to photochemical transformation products, and disruption of chlorophyll function. Effects are strongly influenced by light exposure, turbidity and dissolved organic carbon (DOC), underscoring the importance of environmental conditions during tracer applications. It is therefore recommended to report important endpoint measurements including DOC, pH, water turbidity and temperature.

Data gaps remain for several commonly used dyes, including rhodamine WT and sulforhodamine B, for which algal toxicity data are unavailable.

### 3.2. Invertebrates

Invertebrate responses span a broad range of sensitivities. Among freshwater crustaceans, the beaver-tail fairy shrimp (*Thamnocephalus platyurus*) and water flea (*Daphnia magna*) emerge as comparatively sensitive to rhodamine B, showing impaired survival or growth in short-term assays (Table S1). Other crustaceans, including brine shrimp (*Artemia salina*) and freshwater isopod (*Asellus aquaticus*), exhibit significantly higher tolerance, illustrating pronounced inter-specific variability within this group.

Sulfonated dyes such as sulforhodamine B and rhodamine WT show notably lower toxicity to invertebrates, with endpoints in the table indicating low hazard potential across both freshwater and marine species (Table S1). Fluorescein also exhibits low acute toxicity across crustaceans.

Key methodological limitations include reliance on acute mortality endpoints, limited representation of benthic species, and the infrequent reporting of chronic or reproductive effects. Additionally, many studies do not characterize exposure conditions such as DOC, pH or light regime, limiting comparability.

### 3.3. Fish

Fish generally display higher tolerance to fluorescent dyes than algae and many invertebrates. Across species represented in Table S1 (e.g., zebra fish *Danio rerio*, guppy *Poecilia reticulata*, and rainbow trout *Oncorhynchus mykiss*), rhodamine B appears among the more biologically active dyes, causing growth inhibition or mortality in some acute and extended-duration assays (Table S1). Nevertheless, several fish species demonstrate much lower sensitivity, reflecting differences in life stage, physiology, and exposure duration.

Sulforhodamine B, fluorescein and rhodamine WT show relatively low toxicity to fish, with most endpoints indicating limited hazard at concentrations far exceeding typical environmental exposure during well-designed tracer studies. Marine fish data remain sparse, with most results derived from freshwater species or species tested under mixed salinity (Table S1).

Mechanistic insights mirror patterns seen in other taxa; dyes with higher lipophilicity or photoreactivity tend to induce greater biological effects, while strongly sulfonated dyes exhibit low uptake and low toxicity.

### 3.4. Other Biota

Available data for aquatic plants, mollusks, echinoderms, and amphibians are limited but show similar trends. Marine bivalves and echinoderm embryos exhibit developmental sensitivity to rhodamine B and fluorescein, but generally at concentrations well above those used operationally in tracer tests. Freshwater mollusks (e.g., bloodfluke planorb *Biomphalaria glabrata*) show a broad range of responses, suggesting species-specific tolerance.

Amphibian data are scarce, and most results focus on sub-lethal or developmental endpoints. These taxa are likely to be more responsive to phototoxic mechanisms in shallow, high-light environments, but systematic studies are lacking. Macroalgal and macrophyte data are also limited and often derived from non-standard assays. Overall, the paucity of data for several ecologically important groups underscores the need for broader taxonomic coverage in future assessments.

### 3.5. Inter-Species Comparison

When the toxicity data are considered collectively, several consistent patterns emerge:

- Rhodamine B is the most biologically active dye across taxa, affecting algae, crustaceans and some fish species at the lowest concentrations, relative to other dyes in the dataset. Its higher potency is likely linked to its comparatively low polarity and greater cellular uptake.
- Eosin and related photoreactive dyes show intermediate toxicity, with sensitivity notably higher for some invertebrates.
- Sulfonated dyes (sulforhodamine B, pyranine and sodium naphthionate) show consistently low toxicity across species, reflecting their limited ability to cross biological membranes and their rapid dilution and photodegradation in natural waters.
- Fluorescein shows low acute toxicity across most taxa, though its photolysis products and rapid environmental decay indicate exposure dynamics distinct from rhodamines.
- Taxonomic sensitivity ranks as algae ≥ small crustaceans > fish > mollusks/echinoderms, though exceptions occur (e.g., sensitive shrimp species and developmental stages).

From an operational viewpoint, these findings highlight the importance of selecting dyes with well-characterized and favorable environmental profiles (notably rhodamine WT and sulforhodamine B), minimizing mass release, and accounting for near-field concentration peaks. The results also highlight the need for expanded chronic and sub-lethal testing, the improved reporting of exposure conditions, and evaluation of transformation products, as metabolites can be more toxic than their parent compounds [59]. These are critical gaps for refining environmental protection during tracer use.

## 4. Safe Application

### 4.1. Defining “Safe” Application

Safe application of fluorescent dyes requires balancing hydrological objectives with protection of aquatic life and human health. In regulatory contexts, “safe use” encompasses both the control of environmental exposure and the selection of dyes with well-characterized toxicological profiles. In the USA, rhodamine WT is preferred over rhodamine B because of its lower toxicity and favorable regulatory status [60,61]. Rhodamine B is classified by the USEPA as a List 1 inert ingredient due to carcinogenicity concerns [62] and is identified by NIOSH as a suspected human carcinogen [63]. As a result, its use in environmental tracer studies is generally discouraged.

By contrast, rhodamine WT has been evaluated by the USEPA and USGS for controlled release into natural systems. Its use in public water supply catchments is restricted to brief, infrequent applications that yield finished water concentrations no greater than 0.1 µg/L [60,61]. The term “finished water” [64] refers to treated potable water entering the distribution network; this limit applies after mixing, not at the point of injection. USEPA guidance interprets “brief and infrequent” as individual tracer releases of ≤24 h occurring only a few times per year within a given catchment [65]. In practice, compliance with the 0.1 µg/L finished water limit is achieved through pre-release mass balance modeling, conservative dilution assumptions, and real-time fluorometric monitoring. Planned injection masses are calculated to ensure that predicted concentrations at abstraction points remain at least one order of magnitude below the regulatory threshold under low-flow conditions. Where uncertainty exists, utilities may temporarily suspend abstraction or implement blending strategies to maintain finished water concentrations below the 0.1 µg/L limit.

Other dyes, such as fluorescein, sulforhodamine B, and eosin Y, are used less frequently due to greater toxicity, photolability, or the absence of regulatory evaluation for potable water contexts [3,8,41]. Among commercially available tracers, rhodamine WT remains the only dye with a formalized federal safety framework for environmental hydrological applications.

### 4.2. Critical Analysis of Existing Regulations and Guidelines

Outside of potable water protection, regulatory thresholds for fluorescent dyes are less formalized and typically rely on a combination of ecotoxicological thresholds, aesthetic constraints, and operational guidance. The USGS recommends that dye concentrations remain below levels that cause visible coloration and below 10 µg/L at downstream water user intakes [3]. Kilpatrick and Cobb [3] similarly suggest target peak concentrations of 10–20 µg/L for most surface water applications, balancing detectability with ecological and aesthetic considerations.

In groundwater and karst systems, where attenuation is slow and residence times long, recommended injection concentrations are conservative. Guidance from European and New Zealand tracer practice limits point source concentrations to ≤1 mg/L, ensuring that emergence concentrations at springs or wells remain below 10 µg/L [11].

In estuarine and coastal systems, short-duration tracer studies frequently involve pulse injections where rapid dilution reduces risk. Peak concentrations of 10–20 µg/L are typically achieved at the monitoring site and decline below 10 µg/L within a tidal cycle [3]. Because tidal reflux can prolong exposure, control of release duration is an essential component of safe practice.

Canadian operational guidance [66] mirrors USA recommendations, specifying that dye concentrations should not exceed 10 µg/L outside regulated mixing zones and requiring injections to be positioned to maximize near-field mixing and minimize accumulation in shallow or low-velocity margins.

Together, these guidelines demonstrate broad international agreement: target downstream concentrations should not exceed approximately 10 µg/L for surface water, estuarine, and groundwater systems, except within controlled mixing zones.

### 4.3. Deriving Proposed Application Levels

Table 3 summarizes recommended safe application levels for rhodamine WT, integrating regulatory criteria, ecotoxicological thresholds, environmental attenuation behavior, and operational practice. These values are based on three principles:

- Downstream protection: Target concentrations should remain <10 µg/L at any water user abstraction point or ecological receptor.
- Minimal aesthetic impact: Concentrations should remain below levels of visible coloration.
- Hydrological detection: Concentrations should be high enough to allow for accurate detection by field fluorometers.

These thresholds provide a practical method for environmentally protective and operationally effective dye studies across freshwater, groundwater, and coastal systems.

### 4.4. Application Context and Mixing Zones

In some applications, such as studies of wastewater and industrial outfalls or diffuser systems, initial near-field concentrations may exceed far-field thresholds if rapid dilution occurs. Under the Clean Water Act [64], short-term exceedance of chronic water quality criteria is permissible within a regulated mixing zone. Dye studies often rely on this principle to evaluate mixing efficiency of plume structure over short distances.

For continuous or long duration injections, particularly in tidal and semi-enclosed systems, prediction of steady-state concentrations is essential. The following equation can be used to estimate far-field steady-state concentrations as described in [68]:

$$Css=\frac{C_{0}}{1-r}$$

where

*C_ss_* = predicted far-field steady-state concentration (µg/L);

C_0_ = initial estimated concentration in the far-field;

r = estimated fraction remaining after one tidal cycle.

This equation allows for the pre-release modeling of scenarios where tidal reflux or long residence times may elevate downstream exposure.

In flushing time studies, prediction of concentrations may require the superposition method described by Kilpatrick and Cobb [3] and modified by Goblick et al. [69] which conceptualizes continuous dosing as a cumulative series of repeated, equal-mass slug injections. This method allows for the estimation of the time to reach steady-state and the time required for dye clearance following cessation of injection. These methods support the design of dye studies to meet hydrological objectives without exceeding ecotoxicological thresholds or aesthetic constraints.

## 5. Recommendations for Minimizing Environmental Impacts

The ecotoxicological data presented in Section 3 show that while fluorescent dye tracers are generally safe when used responsibly, their environmental impact is not negligible. Toxicity varies between dye classes, with certain compounds (e.g., rhodamine B and eosin Y) exhibiting acute and chronic effects at concentrations within one to two orders of magnitude of those typically detected in poorly controlled field releases. In addition, phototransformation can increase toxicity in light-exposed surface waters, and short-term exposure near injection points can temporarily exceed biological effect thresholds in areas with low water mixing. The following recommendations provide guidance for the environmentally responsible use of fluorescent tracers in hydrological investigations:

- **Selection of low-toxicity, low-persistence tracers**: Tracers should be selected based on their physico-chemical properties, environmental fate and behavior, and ecotoxicological profile. Dyes with high water solubility, strong sulfonation, low membrane permeability, and rapid photolysis, such as rhodamine WT, sulforhodamine B, and fluorescein sodium, are preferred for most applications. These compounds have LC_50_ and EC_50_ values above 50–100 mg/L across taxa and degrade more rapidly under environmental conditions. In contrast, dyes such as rhodamine B and eosin Y should be avoided in open-channel or high-light environments due to their higher toxicity, photoreactivity, and potential for producing hazardous transformation products. Their use may still be appropriate in confined or subsurface systems (e.g., groundwater tracing) where dilution is rapid and photolytic pathways are absent, but only with careful dose planning.
- **Minimization of injection concentrations and refinement of release protocols**: Where possible, dye mass should be minimized while still achieving detectability. This can be achieved by using fluorometers, conducting pre-tracer simulations or pilot dilutions (under laboratory and/or field conditions) to estimate dispersion and mixing at the injection site, adjusting the injection strategy through selection of slug or continuous release to achieve adequate downstream concentrations without exceeding ecotoxicological thresholds, and ensuring rapid initial mixing (more critical in small streams with incomplete lateral dispersion, where peak dye concentrations along the margins can remain elevated for extended periods).
- **Consideration of light exposure and photodegradation pathways**: Many xanthene dyes photodegrade, forming products with greater toxicity than the parent dye (e.g., [70]). To minimize photodegradation risk, practitioners should avoid photolabile dyes (e.g., rhodamine B and eosin Y) in shallow, clear, high-light environments; use dyes with well-characterized and low-toxicity photoproducts (e.g., fluorescein and rhodamine WT); avoid releases during periods of peak solar irradiance when transformation is the greatest; and whenever possible, select overcast or low-light conditions for surface water injections. These considerations are especially important in oligotrophic and clear water systems.
- **Prevention of localized high-concentration exposure**: Localized exposure immediately downstream of an injection point represents the most significant environmental risk. Harmful peak concentrations can be prevented by pre-mixing dyes in a carrier system, injecting dyes at locations with turbulent, high-energy flows to promote rapid mixing, avoiding injection near sensitive habitats (e.g., macrophyte beds, aquaculture sites, and shallow spawning river beds), and monitoring mixing efficiency in real time with a fluorometer to detect and mitigate stagnation of the dye plume. Where sensitive ecological sites cannot be avoided, ultra-low-toxicity dyes, lower dye masses and continuous injection should be prioritized.
- **Avoidance of cumulative exposure**: Repeated tracer applications within short timeframes can cause cumulative exposure that exceeds that from single injections. To reduce this risk, repeated releases should be time-lagged to allow for complete clearance based on dye persistence data and hydrological residence times. Conservative tracers with short half-lives should be used in studies that require repeated dye releases. Repeated releases of phototoxic dyes should be avoided during periods of low flow or stratification, when dye retention and exposure are greater. This type of cumulative response is more common in environments with limited flushing capacity, such as wetlands, springs, and impoundments.
- **Environmental monitoring during and after tracer studies**: Environmental monitoring provides assurance that dye applications remain within safe concentration levels. Recommended practices include real-time fluorometric monitoring immediately downstream of the injection point to ensure that predicted concentrations are not exceeded, post-injection water quality checks (including physical parameters that could interact with dye behavior, such as pH, temperature, and dissolved organic carbon), and the recording of study conditions (e.g., water flows, weather, and Secchi depth).
- **Integration of ecotoxicological thresholds into study design**: The planning of dye studies should include a formal environmental assessment that integrates acute and chronic toxicity thresholds, comparisons of predicted exposure concentrations with aquatic life benchmarks, and dilution calculations or, in more complex studies, transport modeling to estimate peak and tail concentrations under varying hydrological conditions.
- **Communication planning and compliance**: Environmental protection agencies increasingly require that dye studies be justified, documented, and monitored. Practitioners should provide advance notice to regulatory agencies and affected stakeholders and ensure that risk assessments are documented and emergency response protocols are in place for accidental overdosing, equipment failure, or spills. Regulatory approvals may be required for dye studies, depending on the jurisdiction and national or state regulations.

To improve the practical applicability of the recommendations outlined above, a concise operational framework is proposed to support environmentally protective planning and implementation of fluorescent dye tracer studies (Table 4).

## 6. Conclusions and Research Needs

Fluorescent dyes are essential tools for hydrological investigations, enabling quantitative assessments of transport, mixing, residence times, and hydrological connectivity in both surface and groundwater systems. Their utility originates from their detectability at very low concentrations, relatively low cost, and operational flexibility across a wide range of environmental settings. This literature review consolidates current knowledge on the physico-chemical properties, ecotoxicological profiles, application methods, and safe use of key tracer dyes. Although the available evidence generally indicates that these compounds present low environmental risk when properly applied, several important considerations and research gaps emerge from this review.

First, this review confirms that physico-chemical properties strongly influence the environmental behavior of individual dyes. Differences in environmental behavior translate directly into their ecotoxicological profiles, i.e., dyes characterized by high hydrophilicity and strong ionic character are generally associated with lower toxicity to aquatic organisms. This behavior is attributed not to net molecular charge per se but to limited membrane permeability and low bioaccumulation potential arising from persistent ionization under environmentally relevant conditions. For dyes with pH-dependent ionization, toxicity may vary with environmental conditions that influence speciation. This highlights the importance of ionic behavior rather than nominal charge in determining ecological risk.

Second, while acute toxicity thresholds for most dyes are orders of magnitude above the concentrations typically detected in well-designed dye studies, this safety margin is not uniform. This highlights the importance of tracer selection, mass estimation, and release planning as key factors of environmental safety.

Third, the literature is unequal across different types of dyes and taxa. Evidence on toxicity effects and environmental fate of rhodamine WT, fluorescein, and pyranine is solid, but data for other dyes are lacking. Furthermore, the available toxicity datasets rely heavily on laboratory studies of single species over short exposure durations. Field-based ecological assessments are lacking, and few studies have investigated sub-lethal endpoints such as behavioral alterations and oxidative stress or the combined effects of dyes and their photodegradation products. This evidence is needed to refine best practice guidelines for tracer use and regulatory standards. The priority areas for future research are

- Photo-transformation pathways and toxicity of transformation products.
- Sub-lethal, chronic, and ecosystem-level effects.
- Influence of environmental factors on toxicity and persistence.
- Environmental exposure modeling and risk management.
- Field validation of laboratory toxicity results.
- Assessment of cumulative and repeated exposure.
- Standardization of ecotoxicological testing protocols for tracer studies.

## Figures and Tables

**Table 1:** Physical and chemical properties of fluorescent dyes used in hydrological tracing as sourced from Leibundgut et al. [11].

Commercial Name (Synonyms)	Molecular Formulae (IUPAC Name)	Dye Class	Molecular Weight (g/mol)	Excitation/Emission Maximum Wavelength (nm)
Rhodamine WT (Acid Red 388)	C_29_H_29_N_2_O_5_.Cl. 2Na (ICSC) C_29_H_29_ClN_2_Na_2_O_5_ (PubChem) (disodium;4-[3-(diethylamino)-6-diethylazaniumylidenexanthen-9-yl]benzene-1,3-dicarboxylate;chloride)	Xanthene	567	558/583
Rhodamine B (Basic Violet 10; Brilliant Pink B; Tetraethylrhodamine)	C_28_H_31_N_2_O_3_.Cl (AICIS) C_28_H_31_ClN_2_O_3_ (CAMEO Chemicals; PubChem) ([9-(2-carboxyphenyl)-6-(diethylamino)xanthen-3-ylidene]-diethylazanium;chloride)	Xanthene	479	555/570
Rhodamine 6G (Basic Red 1; C.I. Basic Red 1; Rhodamine F5G)	C_28_H_31_ClN_2_O_3_ (PubChem) ([9-(2-ethoxycarbonylphenyl)-6-(ethylamino)-2,7-dimethylxanthen-3-ylidene]-ethylazanium;chloride)	Xanthene	479	530/552
Fluorescein Sodium (Fluorescein Disodium salt; Soluble Fluorescein; Sodium Fluorescein; Uranin)	C_20_H_10_Na_2_O_5_ (PubChem) (disodium;2-(3-oxido-6-oxoxanthen-9-yl)benzoate)	Xanthene	376.3	490/530
Pyranine (Solvent green 7; Green 8; Japan Green 204; 11,389 Green)	C_16_H_7_Na_3_O_10_S_3_ (PubChem) (trisodium;8-hydroxypyrene-1,3,6-trisulfonate)	Anthraquinone	524.4	405/510
Eosin (Acid Red 87; Eosin yellowish; Eosin Yellowish)	C_20_H_6_Br_4_Na_2_O_5_ (PubChem) (disodium;2-(2,4,5,7-tetrabromo-3-oxido-6-oxoxanthen-9-yl)benzoate)	Xanthene	691.9	525/546
Sodium Naphthionate (Sodium 4-amino-1-naphthalenesulfonate; Naphthionine; Sodium 1-naphthylamine-4-sulfonate; 1-Naphthalenesulfonic acid, 4-amino-, Sodium salt (1:1))	C_10_H_8_NNaO_3_S (PubChem) (sodium;4-aminonaphthalene-1-sulfonate)	Aminonaphthalenesulfonic acid	245.23	325/420

**Table 2:** Characteristics of dye release methods. Information compiled from Kilpatrick and Cobb [3].

Parameter	Pulse (Slug) Injection	Continuous Injection
Main applications	Travel time, contaminant dispersion, hydrological connectivity	Discharge measurement, mixing calibration, steady-state flow characterization
Typical duration of dye release	Minutes to hours	Hours to days
Initial concentration (near release point)	1–100 mg/L	1–50 µg/L
Exposure time	Short-term, transient	Long-term, quasi-steady
Ecological risk	Localized acute exposure possible	Potential for chronic/sub-lethal exposure
Advantages	Simple setup, low total dye volume, high temporal resolution	Stable concentration signal, suited for flow gauging
Limitations	Sensitive to flow variability; rapid decay may reduce detectability	Requires dosing equipment; risk of system malfunction

**Table 3:** Recommended safe application levels for rhodamine WT.

Environment	Target Field Concentration (µg/L)	Release Duration	Reference	Notes
Potable water (finished)	≤0.1 µg/L	≤24 h	[60]	Single-event use only; “brief and infrequent” guidance (USEPA Office of Water)
Streams/rivers	≤10 µg/L	≤24 h	[67]	Avoid visible coloration; USGS field guidance
Groundwater/karst	≤10 µg/L at emergence	Case-dependent (determined by residence time)	[11]	Conservative limit based on persistence and attenuation
Estuarine/coastal	≤10–20 µg/L	≤24 h	[3]	Short-duration tidal pulse studies; rapid dilution expected

Note: Duration reflects residence times in karst systems where breakthrough may persist for weeks; concentration criterion applies at emergence point.

**Table 4:** Operational framework for environmentally protective dye studies.

1. Define objective and hydrological context: Travel time, mixing, discharge, and connectivity; Surface water vs. groundwater vs. estuarine system.
2. Screen dye candidates: Regulatory status; Ecotoxicological profile; Photostability and transformation products; Sorption behavior.
3. Estimate required injection mass: Conservative dilution modeling under low-flow conditions; Target downstream concentration.
4. Evaluate near-field exposure risk: Mixing characteristics; Sensitive habitats; Avoid injection in low-velocity margins.
5. Plan monitoring and contingency: Real-time fluorometry; Pre-defined maximum allowable concentration; Emergency dilution or cessation plan.
6. Post-study verification: Confirm clearance; Document exposure relative to thresholds.

## Data Availability

No new data were created or analyzed in this study. Data sharing is not applicable to this article.

## Supplementary Materials

The following supporting information can be downloaded at: https://www.mdpi.com/article/10.3390/jox16020045/s1, Table S1: Toxicity of fluorescent dyes to aquatic organisms. EC_50_: median effective concentration; LC_50_: median lethal concentration; TL_50_: median tolerance limit.

## Abbreviations

The following abbreviations are used in this manuscript:

DOC	Dissolved Organic Carbon
EC_50_	Median Effective Concentration
LC_50_	Median Lethal Concentration
FDA	Food and Drug Administration
GPS	Global Positioning System
Kow	Octanol–Water Partition Coefficient
NIOSH	National Institute for Occupational Safety and Health
USEPA	United States Environmental Protection Agency
USGS	United States Geological Survey
WT	Water Tracing
