# Supplementary material for: Fluorescent Dyes in Hydrological Tracing: Application Methods, Ecotoxicological Effects, and Safe Application Levels
Source: J Xenobiot. 2026 Mar 3;16(2):45. doi: 10.3390/jox16020045 (PMC13010594; doi:10.3390/jox16020045)
Supplement: Supplementary file 1 [file jox-16-00045-s001.zip › jox-4167583-supplementary.pdf]

Supplementary Material- Journal of Xenobiotics

Fluorescent dyes in hydrological tracing: application methods, ecotoxicological effects, and safe application levels

Carlos J. A. Campos [1,2], Louis A. Tremblay [3*], Olivier Champeau [4], Gregory Goblick [5]

1 Jacobs, 47 Hereford Street Level 2, Wynn Williams Building, Christchurch 8013, Aotearoa New Zealand; carlos.campos@wrcgroup.com
2 Water Research Centre Ltd, Frankland Road, Blagrove, Swindon, Wiltshire, SN5 8YF, UK
3 New Zealand Institute for Bioeconomy Science Ltd, Port Nelson, Nelson 7010, New Zealand; tremblayl@landcareresearch.co.nz
4 Environmental Protection Authority, Wellington 6011, New Zealand
5 Human Foods Program, Office of Laboratory Operations and Applied Science, US Food and Drug Administration, Dauphin Island, AL 36528, USA; Gregory.Goblick@fda.hhs.gov
* Correspondence: tremblayl@landcareresearch.co.nz

Table S1. Toxicity of fluorescent dyes to aquatic organisms. EC50: median effective concentration; LC50: median lethal concentration; TL_{50}: median tolerance limit.

Dye	Test species	Taxon / Common name	Environment	Endpoint	Median or range effect concentration (mg/L) [confidence interval]	Exposure duration	Reference
Rhodamine B	*Raphidocelis subcapitata*	Microalga (Chlorophyta)	Freshwater	Growth inhibition (EC_{50})	14 [8.6–20]	72 h	[14]
Rhodamine B	*Daphnia (Ctenodaphnia) magna*	Crustacean (Arthropoda) / large water flea	Brackish / freshwater	Growth inhibition (EC_{50})	24 [15–32]	48 h	[14]
Rhodamine B	*Danio rerio*	Fish (Chordata) / zebrafish	Freshwater	Growth inhibition (EC_{50})	18 [14–21]	96 h	[14]
Rhodamine B	*Chlorella vulgaris*	Microalga (Chlorophyta) / green alga	Freshwater / terrestrial	IC_{50}	69.94–31.29	24–96 h	[56]
Rhodamine B	*Thamnocephalus platyurus*	Shrimp (Arthropoda) / beavertail fairy shrimp	Freshwater	LC_{50}	8.06	24 h	[55]
Rhodamine B	*Poecilia reticulata*	Fish (Chordata) / guppy	Brackish / freshwater	TL_{50}	24	30 days	[12]
Rhodamine B	*Oncorhynchus mykiss*	Fish (Chordata) / rainbow trout	Marine / brackish / freshwater	LC_{50}	155	96 h	[12]
Rhodamine B	*Oncorhynchus mykiss*	Fish (Chordata) / rainbow trout	Marine / brackish / freshwater	LC_{50}	506	48 h	[12]
Rhodamine B	*Lepomis macrochirus*	Fish (Chordata) / bluegill	Freshwater	LC_{50}	379	96 h	[12]
Rhodamine B	*Ictalurus punctatus*	Fish (Chordata) / channel catfish	Freshwater	LC_{50}	526	96 h	[12]
Rhodamine B	*Asellus (Asellus) aquaticus aquaticus*	Crustacean (Arthropoda) / water hoglouse	Freshwater	LC_{50}	550	96 h	[12]

Rhodamine B	*Artemia salina*	Crustacean (Arthropoda) / brine shrimp	Brackish	LC_{50}	180	24 h	[12]
Rhodamine B	*Daphnia (Ctenodaphnia) magna*	Crustacean (Arthropoda) / large water flea	Brackish / freshwater	LC_{50}	29	72 h	[12]
Rhodamine B	*Crassostrea virginica*	Bivalve (Mollusca) / eastern oyster	Marine	No effect development of eggs	1	48 h	[12]
Rhodamine B	*Hemicentrotus pulcherrimus*	Sea urchin (Echinodermata)	Marine	No effect development of eggs	10	48 h	[12]
Rhodamine B	*Mytilus edulis*	Bivalve (Mollusca) / blue mussel	Marine	No effect development of eggs	3.2	48 h	[12]
Rhodamine B	*Corbicula manilensis*	Bivalve (Mollusca) / Asiatic clam	Freshwater	LC_{50}	>500	96 h	[12]
Rhodamine B	*Biomphalaria glabrata*	Snail (Mollusca) / bloodfluke planorb	Freshwater	LC_{50}	1–10	120 h	[12]
Sulforhodamine B	*Poecilia reticulata*	Fish (Chordata) / guppy	Brackish / freshwater	TL_{50}	58–116	30 days	[12]
Sulforhodamine B	*Oncorhynchus mykiss*	Fish (Chordata) / rainbow trout	Marine / brackish / freshwater	LC_{50}	450	48 h	[12]
Sulforhodamine B	*Oryzias latipes*	Fish (Chordata) / Japanese rice fish	Brackish / freshwater	LC_{50}	>3,000	48 h	[12]
Sulforhodamine B	*Daphnia (Ctenodaphnia) magna*	Crustacean (Arthropoda) / large water flea	Brackish / freshwater	LC_{50}	139	72 h	[12]
Fluorescein	*Poecilia reticulata*	Fish (Chordata) / guppy	Brackish / freshwater	TL_{50}	752	30 days	[12]
Fluorescein	*Oncorhynchus mykiss*	Fish (Chordata) / rainbow trout	Marine / brackish / freshwater	LC_{50}	1,372	96 h	[12]
Fluorescein	*Oncorhynchus mykiss*	Fish (Chordata) / rainbow trout	Marine / brackish / freshwater	LC_{50}	3,420	48 h	[12]

Fluorescein	*Lepomis macrochirus*	Fish (Chordata) / bluegill	Freshwater	LC_{50}	3,433	96 h	[12]
Fluorescein	*Ictalurus punctatus*	Fish (Chordata) / channel catfish	Freshwater	LC_{50}	2,267	96 h	[12]
Fluorescein	*Oryzias latipes*	Fish (Chordata) / Japanese rice fish	Brackish / freshwater	LC_{50}	3,000	48 h	[12]
Fluorescein	*Artemia salina*	Crustacean (Arthropoda) / brine shrimp	Brackish	LC_{50}	100–300	24 h	[12]
Fluorescein	*Daphnia (Ctenodaphnia) magna*	Crustacean (Arthropoda) / large water flea	Brackish / freshwater	LC_{50}	165	72 h	[12]
Fluorescein	*Hemicentrotus pulcherrimus*	Sea urchin (Echinodermata)	Marine	No effect development of eggs	10	48 h	[12]
Fluorescein	*Mytilus edulis*	Bivalve (Mollusca) / blue mussel	Marine	No effect development of eggs	1	48 h	[12]
Fluorescein	*Biomphalaria glabrata*	Snail (Mollusca) / bloodfluke planorb	Freshwater	LC_{50}	1–10	120 h	[12]
Rhodamine WT	*Poecilia reticulata*	Fish (Chordata) / guppy	Brackish / freshwater	TL_{50}	1,360	30 days	[12]
Rhodamine WT	*Oncorhynchus mykiss*	Fish (Chordata) / rainbow trout	Marine / brackish / freshwater	LC_{50}	>320	96 h	[12]
Rhodamine WT	*Oncorhynchus mykiss*	Fish (Chordata) / rainbow trout	Marine / brackish / freshwater	LC_{50}	>320	48 h	[12]
Rhodamine WT	*Asellus (Asellus) aquaticus aquaticus*	Crustacean (Arthropoda) / water hoglouse	Freshwater	LC_{50}	>2,000	96 h	[12]
Rhodamine WT	*Daphnia (Ctenodaphnia) magna*	Crustacean (Arthropoda) / large water flea	Brackish / freshwater	LC_{50}	170	72 h	[12]
Rhodamine WT	*Magallana gigas*	Bivalve (Mollusca) / Pacific oyster	Marine / brackish	LC_{50}	10	48 h	[12]
Rhodamine WT	*Thamnocephalus platyurus*	Shrimp (Arthropoda) / beavertail fairy shrimp	Freshwater	LC_{50}	1,698	24 h	[12]

Eosin	*Poecilia reticulata*	Fish (Chordata) / guppy	Brackish / freshwater	TL_{50}	138	30 days	[12]
Eosin	*Oncorhynchus mykiss*	Fish (Chordata) / rainbow trout	Marine / brackish / freshwater	LC_{50}	>100	96 h	[12]
Eosin	*Oncorhynchus mykiss*	Fish (Chordata) / rainbow trout	Marine / brackish / freshwater	LC_{50}	>100	48 h	[12]
Eosin	*Oryzias latipes*	Fish (Chordata) / Japanese rice fish	Brackish / freshwater	LC_{50}	1,800	48 h	[12]
Eosin	*Daphnia (Ctenodaphnia) magna*	Crustacean (Arthropoda) / large water flea	Brackish / freshwater	LC_{50}	90	72 h	[12]
Sodium naphtionate	*Daphnia (Ctenodaphnia) magna*	Crustacean (Arthropoda) / large water flea	Brackish / freshwater	EC_{50}	2,791	48 h	[20]
Sodium naphtionate	*Raphidocelis subcapitata*	Microalga (Chlorophyta)	Freshwater	EC_{50}	63.2	72 h	[20]

References:

12. Smart, P. A review of the toxicity of twelve fluorescent dyes used for water tracing. *National Speleological Society Bulletin* **1984**, *46*, 21–33.
14. Skjolding, L.; Jorgensen, L.; Dyhr, K.; Köppl, C.; McKnight, U.; Bauer-Gottwein, P.; Mayer, P.; Bjerg, P.; Baun, A. Assessing the aquatic toxicity and environmental safety of tracer compounds Rhodamine B and Rhodamine WT. *Water Research* **2021**, *197*, doi:10.1016/j.watres.2021.117109.
20. Gombert, P.; Biaudet, H.; de Seze, R.; Pandard, P.; Carré, J. Toxicity of fluorescent tracers and their degradation byproducts. *International Journal of Speleology* **2017**, *46*, 23–31, doi:10.5038/1827-806x.46.1.1995.
55. Rowinski, P.; Chrzanowski, M. Influence of selected fluorescent dyes on small aquatic organisms. *Acta Geophysica* **2011**, *59*, 91–109, doi:10.2478/s11600-010-0024-7.
56. Sudarshan, S.; Bharti, V.; Harikrishnan, S.; Shukla, S.; RathiBhuvaneswari, G. Eco-toxicological effect of a commercial dye Rhodamine B on freshwater microalgae Chlorella vulgaris. *Archives of Microbiology* **2022**, *204*, doi:10.1007/s00203-022-03254-5.
